# Pharmacological inhibition of TAK1 prevents and induces regression of experimental organ fibrosis

**DOI:** 10.1172/jci.insight.165358

**Published:** 2023-07-24

**Authors:** Swarna Bale, Priyanka Verma, Bharath Yalavarthi, Scott Arthur Scarneo, Philip Hughes, M. Asif Amin, Pei-Suen Tsou, Dinesh Khanna, Timothy A.J. Haystead, Swati Bhattacharyya, John Varga

**Affiliations:** 1 Michigan Scleroderma Program, Division of Rheumatology, Department of Internal Medicine, University of Michigan, Ann Arbor, Michigan, USA.; 2 EydisBio Inc., Durham, North Carolina, USA.; 3 Department of Pharmacology and Cancer Biology, Duke University School of Medicine, Durham, North Carolina, USA.

**Keywords:** Autoimmunity, Dermatology, Extracellular matrix, Fibronectin, Fibrosis

## Abstract

Multiorgan fibrosis in systemic sclerosis (SSc) accounts for substantial mortality and lacks effective therapies. Lying at the crossroad of TGF-β and TLR signaling, TGF-β–activated kinase 1 (TAK1) might have a pathogenic role in SSc. We therefore sought to evaluate the TAK1 signaling axis in patients with SSc and to investigate pharmacological TAK1 blockade using a potentially novel drug-like selective TAK1 inhibitor, HS-276. Inhibiting TAK1 abrogated TGF-β1 stimulation of collagen synthesis and myofibroblasts differentiation in healthy skin fibroblasts, and it ameliorated constitutive activation of SSc skin fibroblasts. Moreover, treatment with HS-276 prevented dermal and pulmonary fibrosis and reduced the expression of profibrotic mediators in bleomycin-treated mice. Importantly, initiating HS-276 treatment even after fibrosis was already established prevented its progression in affected organs. Together, these findings implicate TAK1 in the pathogenesis of SSc and identify targeted TAK1 inhibition using a small molecule as a potential strategy for the treatment of SSc and other fibrotic diseases.

## Graphical abstract

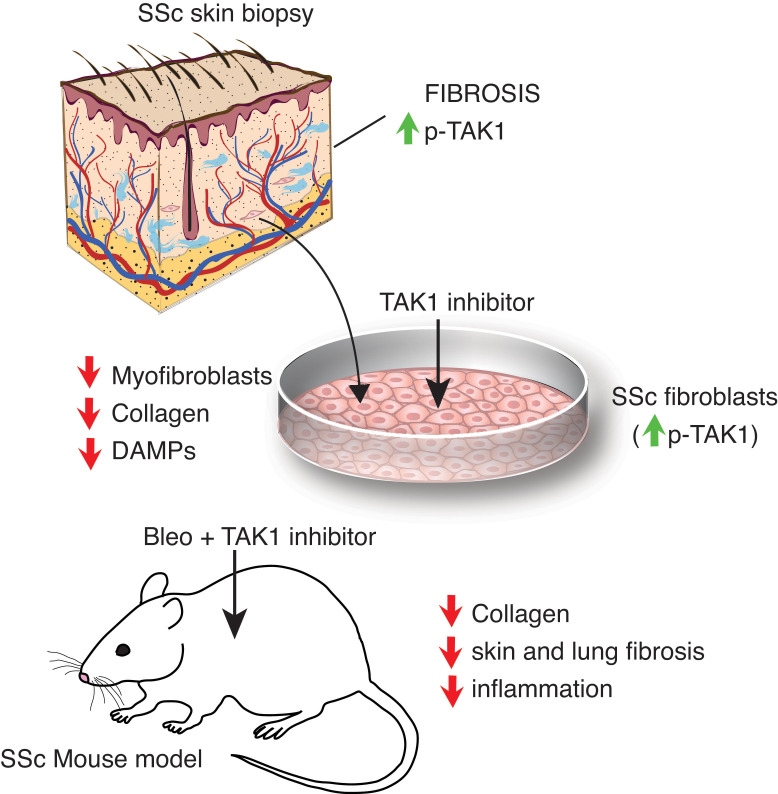

## Introduction

The pathogenesis of systemic sclerosis (SSc) involves vasculopathy, immune dysregulation, and aberrant tissue fibrosis in skin and multiple internal organs (1, 2). TGF-β has potent profibrotic activity, and aberrant TGF-β activity is implicated in SSc pathogenesis (3). Excessive production and tissue accumulation of extracellular matrix (ECM) components is a hallmark of SSc (4). The TGF-β–activated kinase 1 (TAK1), a member of the mitogen-activated protein kinase (MAP kinase kinase kinase [MAP3K]) family, mediates noncanonical TGF-β signaling (5, 6). Moreover, TAK1 has also been implicated as a critical node in TLR-dependent signaling via NF-κB (7). In this respect, it has been shown that TLR4/TAK1 promotes inflammation (8), while inhibition of this pathway blocks agonist activation and reduces expression of downstream proinflammatory mediators (9–13). We previously demonstrated that endogenous TLR ligand damage-associated molecular patterns (DAMPs), including fibronectin-extra domain A (Fn-EDA) and tenascin-C, trigger TLR4-dependent fibrotic responses implicated in SSc (14, 15). In view of the involvement of TAK1 in multiple types of profibrotic responses, it is not surprising that, in mice, targeted genetic ablation of TAK1 in kidney, lung, and skeletal muscle were associated with protection from fibrosis and inflammation (16–19). Importantly, adult mice with fibroblast-specific deletion of TAK1 showed delayed dermal wound repair (20). Moreover, embryonic mouse fibroblasts lacking TAK1 showed impaired TGF-β–dependent profibrotic signaling (21). Together, these observations suggest that TAK1 might be implicated in SSc pathogenesis and that pharmacological targeting of TAK1 might represent a viable treatment strategy to ameliorate SSc as well as other form of fibrosis.

Inhibitors of TAK1 have been developed for the treatment of various conditions, but their development for clinical application has stalled due to suboptimal selectivity and bioavailability (22, 23). Recently, using a directed medicinal chemistry approach, we developed a highly selective and potent TAK1 inhibitor (13). The potentially novel TAK1 inhibitor (HS-276) is a small molecule with low nM affinity (IC_50_ = 2.5 nM) toward TAK1 and is orally bioavailable (>95% bioavailability with μM plasma levels) (13). Here we sought to explore the role of TAK1 in the pathogenesis of SSc and to evaluate the effect of the potentially novel TAK1 inhibitor in fibroblasts and in experimental models of dermal and pulmonary fibrosis. Our results demonstrate that HS-276 treatment initiated concurrently with bleomycin, or subsequent to bleomycin, both mitigated the severity of dermal and pulmonary fibrosis in mice. Notably, HS-276 attenuated collagen synthesis and myofibroblasts differentiation in constitutively active SSc fibroblasts as well as in TGF-β1–treated healthy skin fibroblasts. Taken together, our findings provide robust experimental support for the pathogenic role of TAK1 in SSc and suggest that selective TAK1 inhibition using a novel small molecule might be an attractive treatment strategy for SSc and other fibrotic diseases.

## Results

### TAK1 is activated in SSc, and its selective inhibitor HS-276 ameliorated constitutive fibroblast activation.

**Figure 1:**
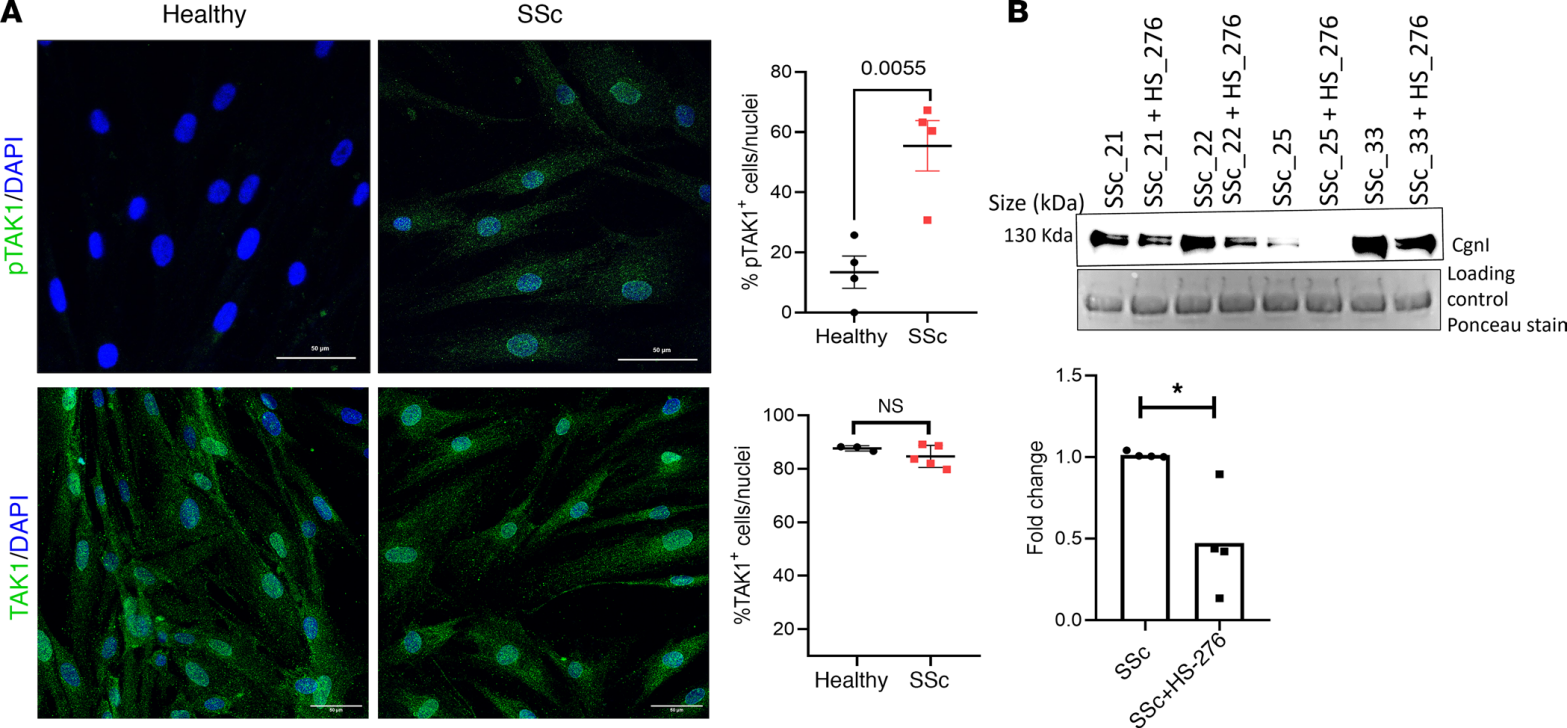
TAK1 is activated in SSc. (**A**) Skin fibroblasts from patients with SSc (*n* = 4) and healthy controls (*n* = 4) at confluence were immunolabeled with antibodies to phospho-TAK1 (top panel) and TAK1 (bottom panel); immunopositive cells (means from 3 randomly selected regions) were quantified. Unpaired *t* test. (**B**) Secreted collagen I (cgnI) in culture media from SSc fibroblasts (*n* = 4) with or without HS-276 treatment. Top panel: immunoblots. Bottom panel: quantification of cgnI. *P < 0.05.

To examine TAK1 activation in SSc, we sought to determine the levels of phosphorylated TAK1 in explanted SSc and healthy control skin fibroblasts and in skin biopsies from patients with SSc and healthy controls. Significant TAK1 activation was observed in explanted SSc skin fibroblasts (*P* = 0.0055) and in SSc skin biopsies (3.81-fold increase), compared with healthy control fibroblasts and skin biopsies (Figure 1A and Supplemental Figure 1A; supplemental material available online with this article; https://doi.org/10.1172/jci.insight.165358DS1). There was no significant difference in total TAK1 levels in SSc fibroblasts compared with healthy fibroblasts (Figure 1A, lower panel). To explore TAK1 activation in SSc-associated interstitial lung disease (SSc-ILD), a frequent and potentially deadly complication of SSc (24), we measured phospho-TAK1 in lung tissue from patients with SSc-ILD and controls without SSc. Compared with control lungs, strong phospho-TAK1 expression was noted in each SSc-ILD lung examined (Supplemental Figure 1B).

**Figure 2:**
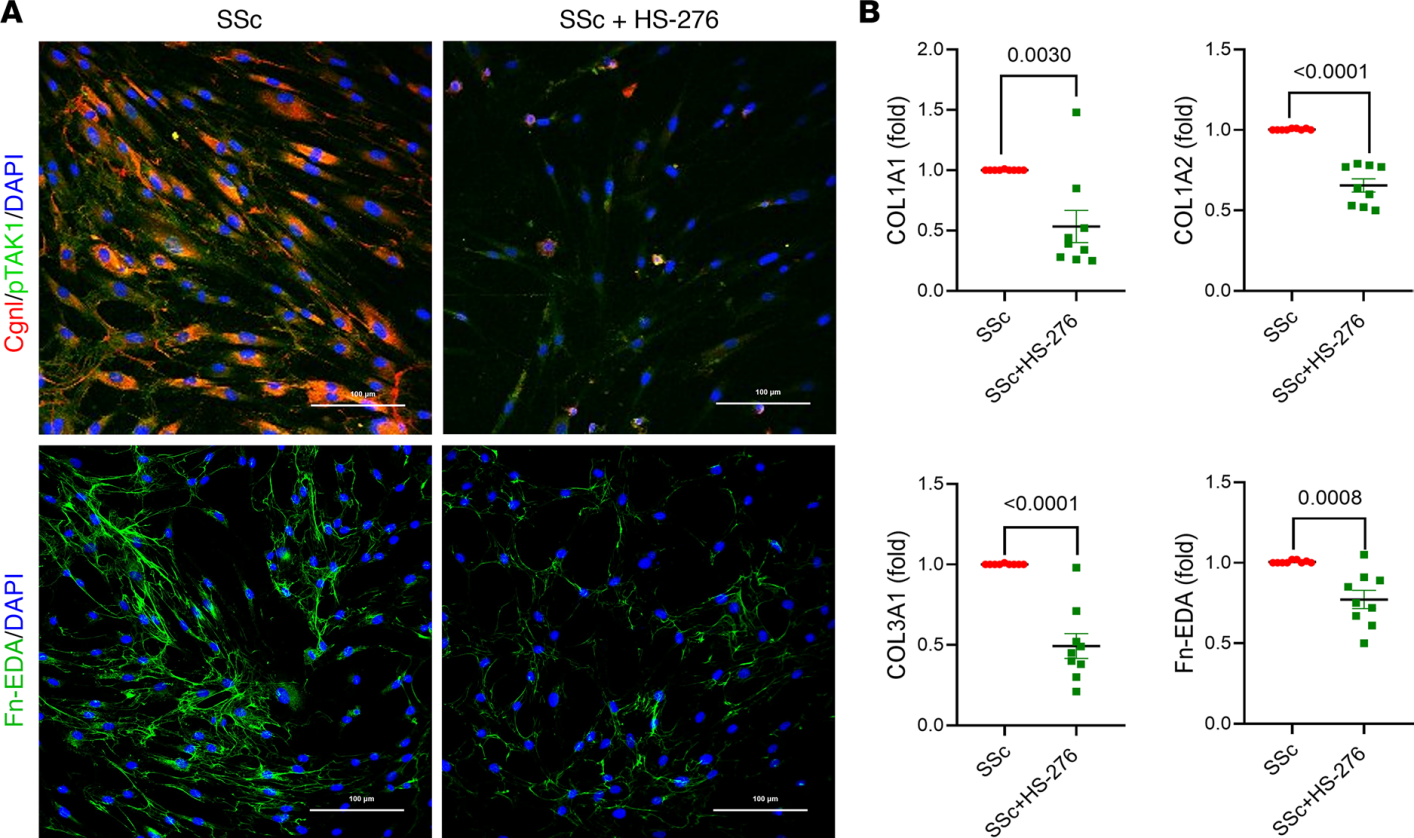
HS-276 reduced ECM deposition and TAK1 activation in SSc fibroblasts. SSc skin fibroblasts were cultured with or without HS-276 treatment for 24 hours. (**A**) Fibroblasts were immunolabeled using antibodies to phospho-TAK1 and type I collagen (*n* = 4). Scale bar: 100 μm (top panel). Fibroblasts were immunolabeled using antibodies to Fn-EDA (*n* = 6). Scale bar: 100 μm (bottom panel). Representative images. (**B**) Confluent SSc skin fibroblasts (*n* = 9) were incubated with HS-276 for 24 hours, and mRNA levels were quantitated by qPCR. Unpaired *t* test.

We next sought to evaluate the effects of TAK1 inhibition on fibrotic gene expression in skin fibroblasts isolated from patients with SSc. Explanted SSc fibroblasts treated with HS-276 displayed significant reduction of secreted collagen I (Figure 1B) and cellular collagen I (*P* < 0.0001) as well as Fn-EDA (*P* = 0.0039) (Figure 2A and Supplemental Figure 2B). These antifibrotic responses were accompanied by downregulation of phospho-TAK1 (*P* = 0.0416; Figure 2A and Supplemental Figure 1B). Consistently, HS-276 significantly reduced the expression of *COL1A1* (*P* = 0.003), *COL1A2* (*P* < 0.0001), and *COL3A1* (*P* < 0.0001) expression (Figure 2B) and attenuated the elevated expression of the profibrotic DAMPs Fn-EDA (Figure 2B, *P* = 0.0008) and tenascin-C (Supplemental Figure 2A, *P* < 0.0001).

### HS-276 inhibits TGF-β–induced cellular fibrotic responses.

**Figure 3:**
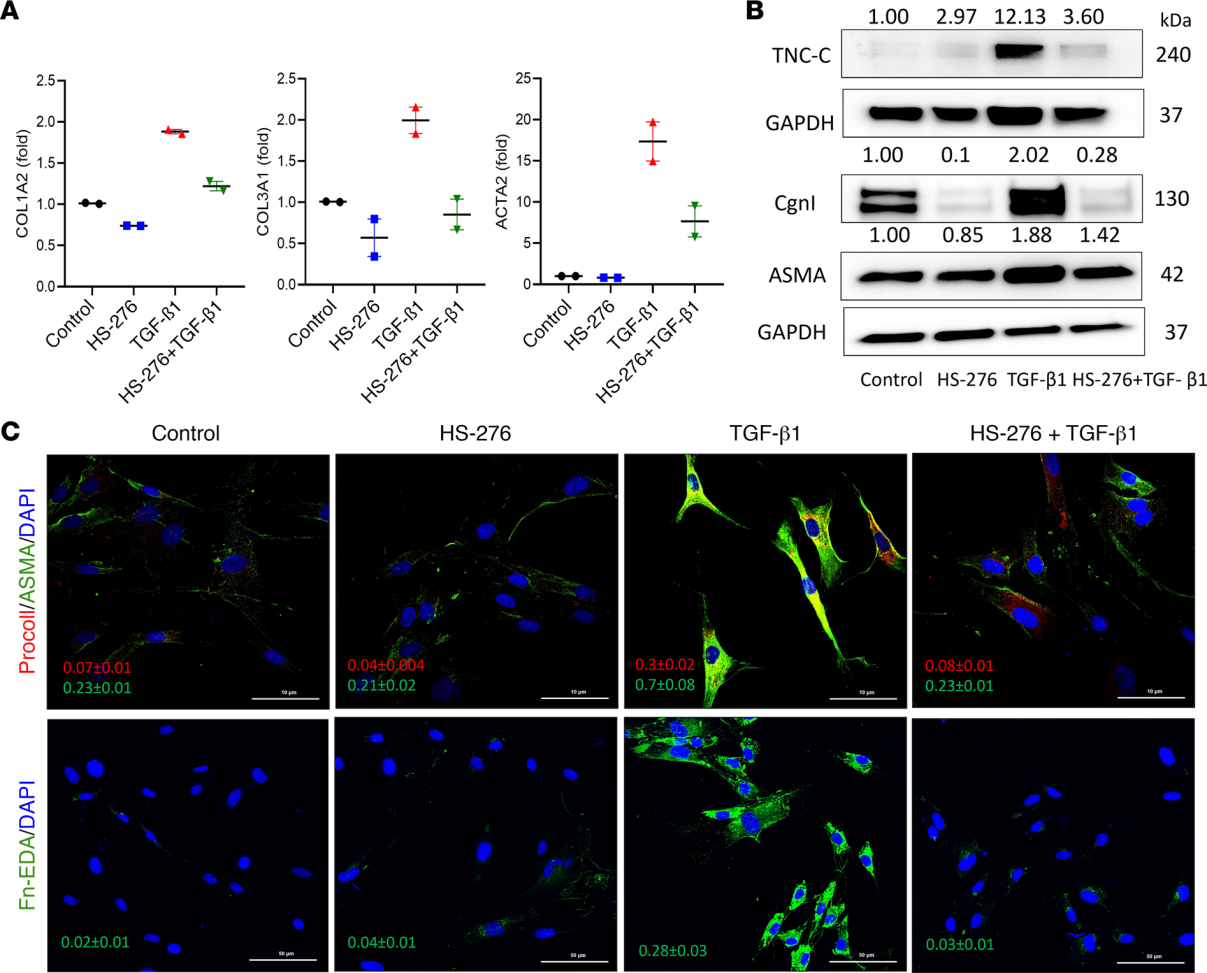
HS-276 inhibited TGF-β–dependent fibrotic cellular responses. Confluent foreskin fibroblasts were incubated with TGF-β1 (10 ng/mL) for 24 hours in the presence or absence of HS-276 (10 μM). (**A**) qPCR. Levels were normalized with GAPDH. (**B**) Whole-cell lysates examined by immunoblotting; representative immunoblots. Relative fold change compared with control, normalized with GAPDH. (**C**) Fibroblasts were immunolabeled using antibodies to procollagen I, ASMA, or Fn-EDA. Representative images. Scale bar: 10 μm (top panel) or 50 μm (bottom panel). Quantification of relative fluorescence intensities (means ± SEM from 3 randomly selected regions).

**Figure 4:**
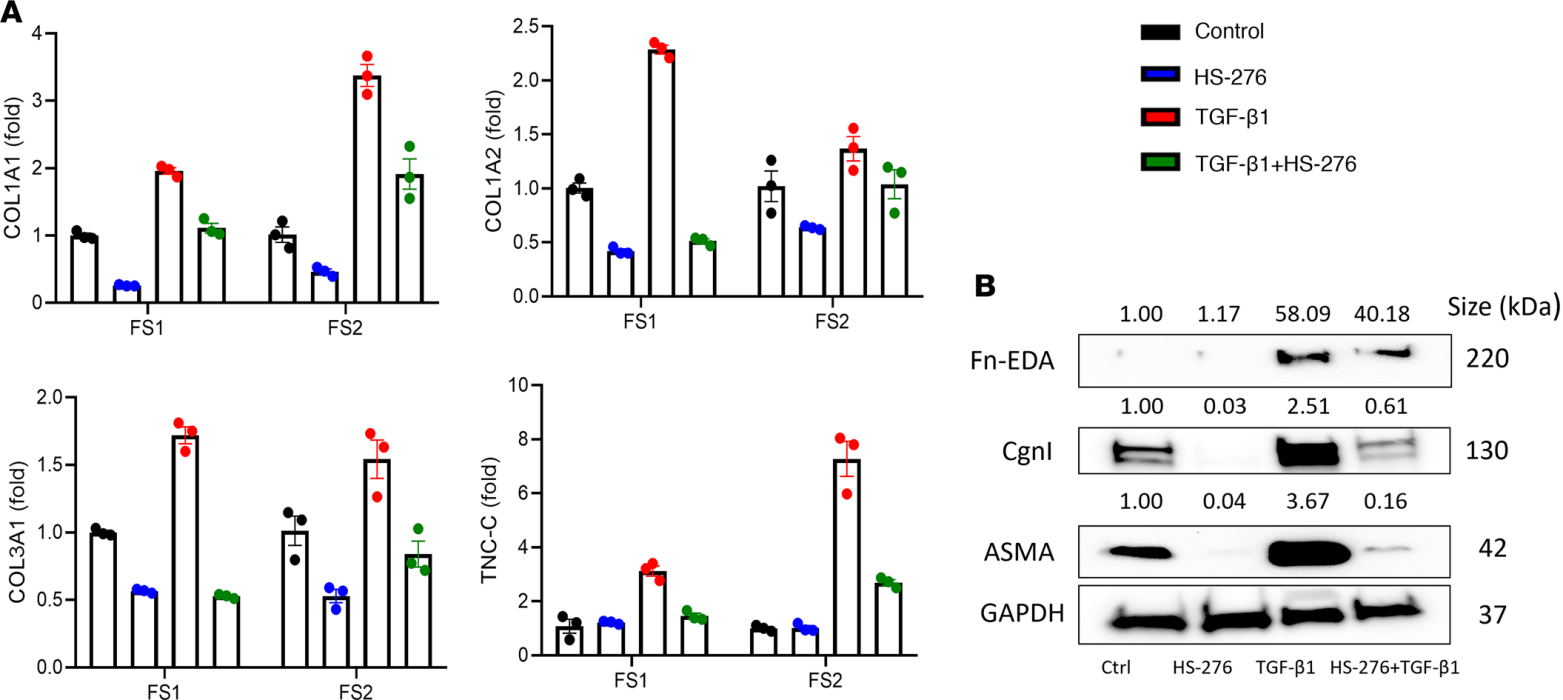
HS-276 reverses TGF-β–induced fibrotic responses. Confluent foreskin fibroblasts were incubated with or without TGF-β1 for 24 hours, followed by addition of HS-276 (10 μM) for a further 24 hours. (**A**) mRNA levels determined by qPCR (*n* = 2 biological replicates, 2 independent foreskin fibroblasts [FS]). (**B**) Whole-cell lysates subjected to immunoblotting. Representative immunoblots with relative fold change compared with control, normalized with GAPDH.

Next, we examined the effect of HS-276 on TGF-β1–induced profibrotic responses using human foreskin fibroblasts and healthy adult skin fibroblasts. Following treatment with HS-276, TGF-β1–treated fibroblasts showed marked reduction in *COL1A2*, *COL3A1*, and *ACTA2* expression (Figure 3A and Supplemental Figure 3). Furthermore, HS-276 blocked TGF-β1–induced upregulation of α smooth muscle actin (α-SMA), collagen I, Fn-EDA, and tenascin-C (Figure 3, B and C). Comparable antifibrotic effects of HS-276 were observed in healthy adult skin fibroblasts (Supplemental Figure 4). Notably, despite its potent effects on TGF-β–dependent fibrotic cellular responses, TAK1 inhibition failed to prevent Smad2 phosphorylation (*P* = 0.944) in TGF-β1–stimulated fibroblasts, indicating that the antifibrotic effects were Smad independent. There were no changes in total Smad2 expression in fibroblasts treated with HS-276 (Supplemental Figure 5). We next evaluated the effects of TAK1 inhibition on TGF-β1–mediated upregulation of fibrotic genes. In fibroblasts preincubated with TGF-β1 for 24 hours, addition of HS-276 attenuated profibrotic cellular responses (Figure 4). We used global transcriptome analysis to evaluate genome-wide effects of TAK1 inhibition. Compared with fibroblasts treated with TGF-β alone, fibroblasts treated with TGF-β plus HS-276 showed downregulation of multiple inflammatory genes; furthermore, GO pathway analysis demonstrated attenuation of several fibrosis-associated pathways (Supplemental Figure 6).

### HS-276 ameliorated skin and lung fibrosis in mice.

**Figure 5:**
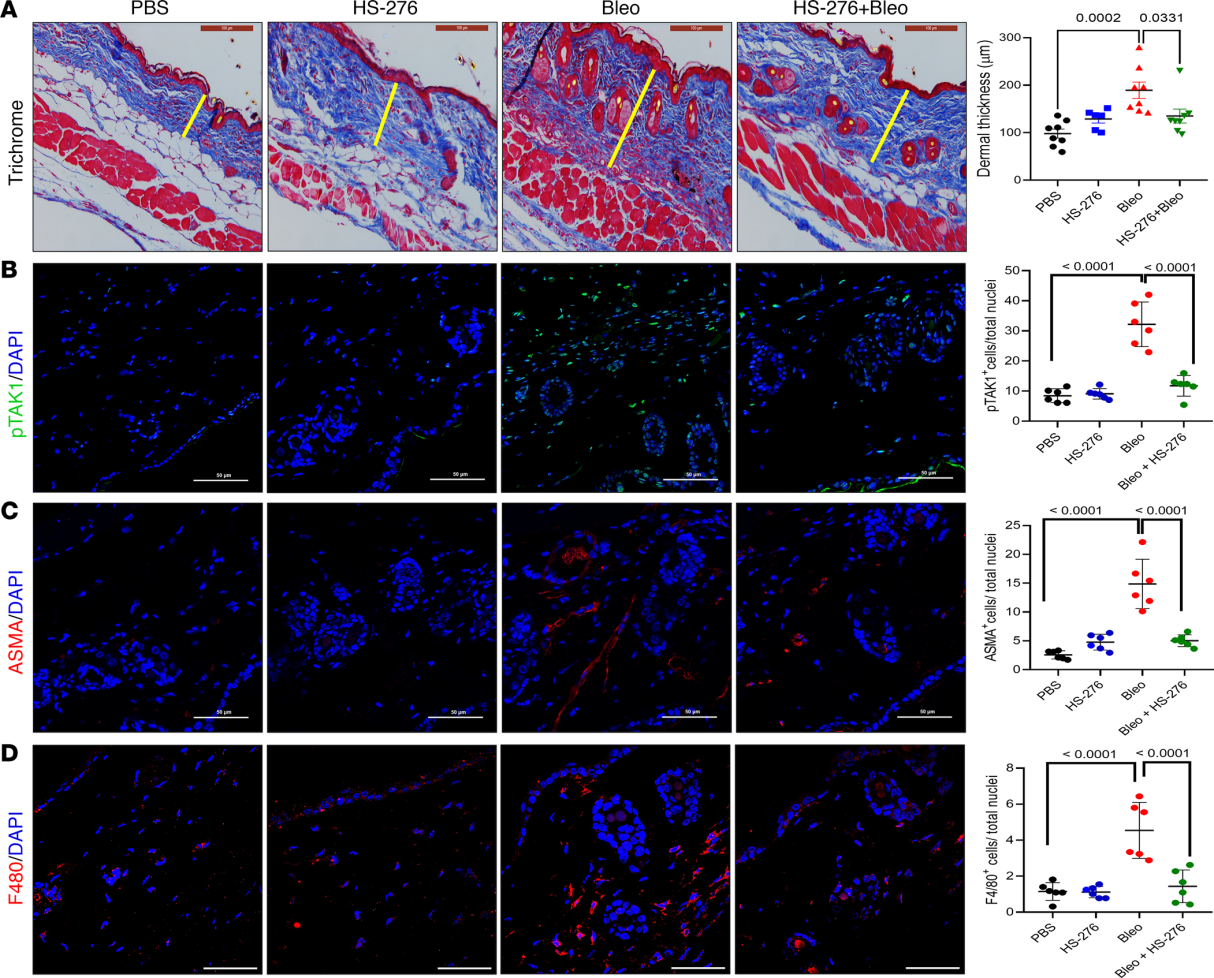
HS-276 treatment prevented bleomycin-induced skin fibrosis. C57/BL6 mice were randomized to 4 treatment groups (*n* = 6–8 mice/group), euthanized on day 22, and skin was harvested. (**A**) Trichrome stains, representative images. Scale bar: 100 μm (left panel). Dermal thickness (right panel, means ± SEM of 8 determinations/hpf). One-way ANOVA followed by Tukey’s multiple-comparison test. (**B**–**D**) Skin sections were immunolabeled with antibodies to phospho-TAK1 (green; scale bar: 50 μm), ASMA (red; scale bar: 50 μm), F4/80 (red; scale bar: 10 μm), and DAPI (blue). Representative images (left panel). Quantification of immunopositive cells (right panel; means from 3 randomly selected regions from 3 mice/group). One-way ANOVA followed by Tukey’s multiple-comparison test.

We next sought to evaluate the impact of HS-276 treatment in a murine model of SSc. To induce skin and lung fibrosis, C57BL/6J mice were administered s.c. bleomycin injections (10 mg/kg) daily for 2 weeks (5 days/week) concurrently with HS-276 (25 mg/kg) or vehicle administered by daily i.p. injections (5 days/week). No signs of toxicity or behavioral changes were observed with HS-276 treatment. Mice were sacrificed at day 22, and lesional skin and lung were harvested. The thickness of the dermis, markedly increased in bleomycin-treated mice (*P* < 0.001), was significantly reduced when HS-276 was administered concomitantly with bleomycin (*P* = 0.033) (Figure 5A). Moreover, bleomycin-induced attrition of intradermal white adipose tissue accompanying skin fibrosis was also attenuated with HS-276 treatment (Figure 5A). A marked increase in phospho-TAK1 expression in the dermis, observed in bleomycin-injected mice, was fully abrogated with HS-276 treatment (Figure 5B, *P* < 0.0001).

Moreover, HS-276 in bleomycin-treated mice significantly attenuated the increase in dermal collagen deposition (*P* = 0.042) and expression of *ACTA2* (*P* = 0.0047) and *TGFB1* (*P* = 0.0148) genes (Supplemental Figure 7, A and B); it also reduced the numbers of α-SMA^+^ interstitial myofibroblasts (*P* < 0.0001) and F4/80^+^ macrophages (*P* < 0.0001) within the lesional skin (Figure 5, C and D).

**Figure 6:**
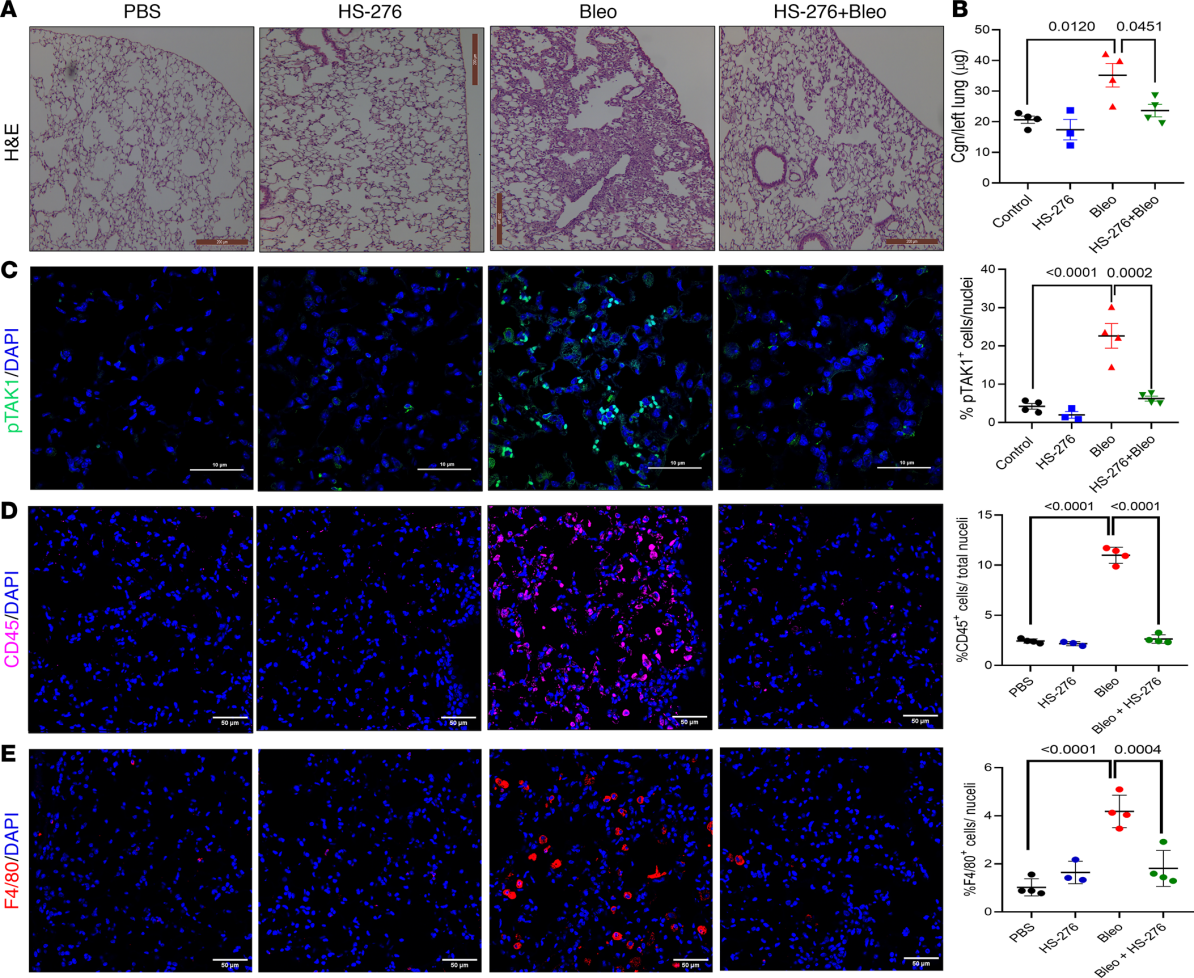
HS-276 inhibited bleomycin-induced lung fibrosis. C57BL/6 mice were randomized to 4 treatment groups and euthanized on day 22, and lungs were harvested. (**A**) H & E stain. Representative images. Scale bar: 200 μm. (**B**) Hydroxyproline assays, left lungs (*n* = 3–4). Results are mean ± SEM. One-way ANOVA followed by Tukey’s multiple-comparison test. (**C**–**E**) Lung sections were immunolabeled with antibodies to phospho-TAK1 (scale bar: 10 μm), CD45 (scale bar: 50 μm), F4/80 (scale bar: 50 μm), and DAPI (blue). Representative images (left panel); quantification of immunopositive cells (right panel, means from 3 randomly selected regions from 4 mice/group). One-way ANOVA followed by Tukey’s multiple-comparison test.

In view of the pronounced TAK1 activation observed in SSc-ILD lungs (Supplemental Figure 1B), subsequent experiments sought to explore the effect of TAK1 inhibition on lung fibrosis. S.c. bleomycin injections elicited prominent pulmonary architectural changes, with influx of inflammatory cells accompanied by presence of fibrotic foci primarily in the subpleural area, along with perivascular and interstitial fibrosis (Figure 6A). These pathological changes in the lungs were associated with significant increase in collagen accumulation (*P* = 0.012) and TAK1 activation (*P* < 0.0001) (Figure 6, B and C), while a marked reduction of collagen deposition by HS-276 treatment was observed (Supplemental Figure 7, C and D). Furthermore, HS-276 attenuated the increase in inflammatory markers CD45 (*P* < 0.0001) and F4/80 (*P* = 0.0004) in the lung (Figure 6, D and E).

### Treatment with HS-276 initiated after emergence of fibrosis prevented its further progression in the skin and lung.

**Figure 7:**
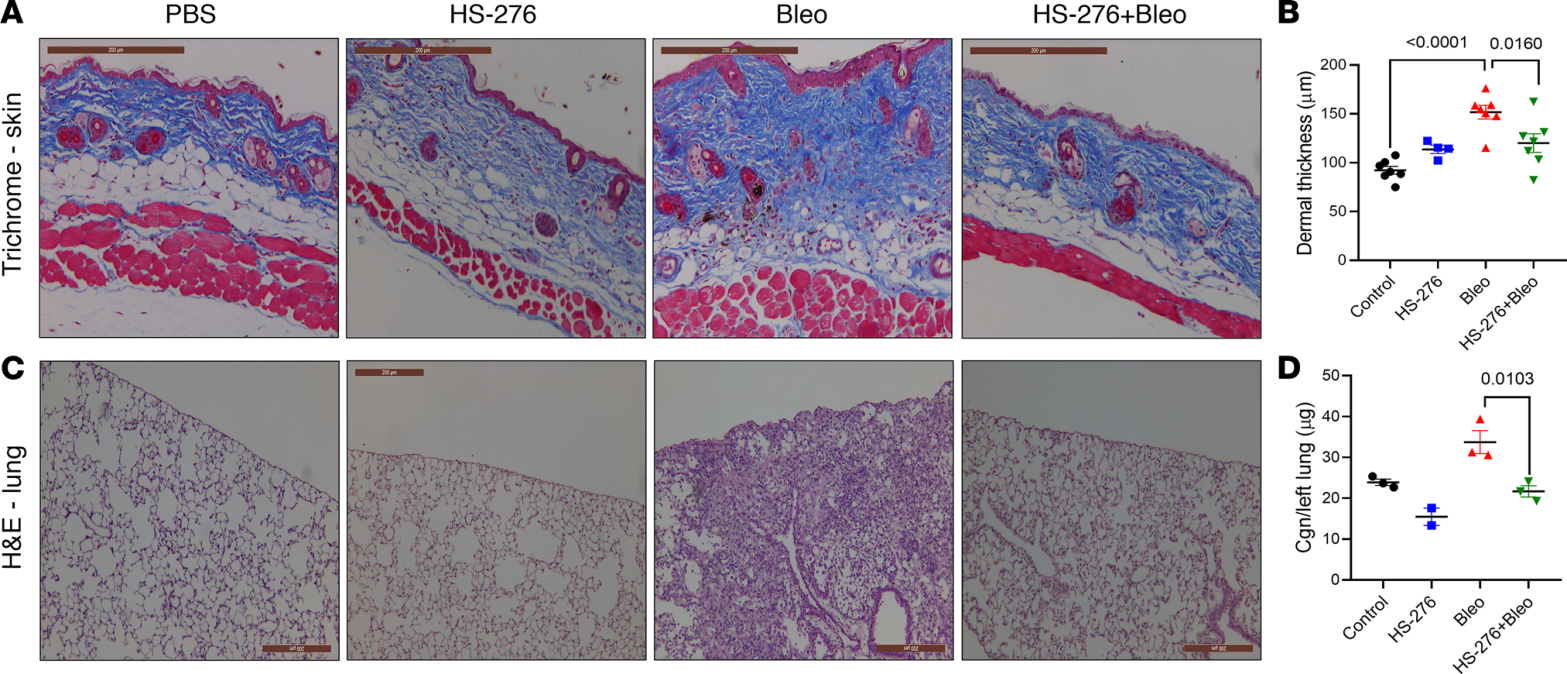
HS-276 treatment mitigated established skin and lung fibrosis. C57BL/6 mice were given daily s.c. injections of PBS or bleomycin, and HS-276 (25 mg/kg) via daily i.p. injections were started on day 14. They continued until day 28, when mice were euthanized, and skin and lungs were harvested for analysis. (**A**) Trichrome stains of skin, representative images. Scale bar:200 μm. (**B**) Dermal thickness (means ± SEM of 8 determinations/hpf, *n* = 4–7). One-way ANOVA followed by Tukey’s multiple-comparison test. (**C**) H&E stains of lung sections, representative images. Scale bar: 200 μm. (**D**) Hydroxyproline assays, left lung (*n* = 2–3). One-way ANOVA followed by Tukey’s multiple-comparison test.

To further investigate the modulation of fibrosis by TAK1, we next evaluated whether delayed TAK1 inhibition will ameliorate bleomycin-induced skin and lung changes. In these experiments, fibrosis was induced by 10 injections of bleomycin over 14 days, followed by 14 days of daily HS-276 (25 mg/kg, i.p.) or vehicle treatment. At 28 days after bleomycin induction, animals were sacrificed, and skin and lungs were removed for analysis. Treatment with HS-276 dramatically attenuated bleomycin-induced increase in dermal thickness (*P* = 0.016) and loss of dermal white adipose tissue (Figure 7, A and B) — as well as the influx of inflammatory cells and subpleural, perivascular, and interstitial fibrosis and collagen accumulation (*P* = 0.0103) (Figure 7, C and D) — demonstrating the potential of HS-276 to prevent the progression of established fibrosis. Schematic illustration for the proposed mechanisms underlying the beneficial effects of TAK1 inhibition is shown in Supplemental Figure 8.

## Discussion

The pathogenesis of SSc is incompletely understood, and the disease remains associated with high mortality (2, 25). At present, no effective treatments exist; therefore, there is an urgent need to develop improved therapies (26, 27). Because aberrant TGF-β expression is implicated in the pathogenesis of SSc, TGF-β represents a potential therapeutic target (28). However, blocking TGF-β activity might lead to spontaneous immune activation, epithelial hyperplasia, impaired wound healing, and other adverse effects (29). Previous reports have implicated TAK1 in both noncanonical TGF-β as well as TLR4-dependent signaling (30–32). Furthermore, TAK1 has been shown to serve as a critical mediator of fibroinflammatory responses (18, 33–35). Based on these observations, we hypothesized that blockade of TAK1 might mitigate SSc fibrosis and represent a novel therapeutic strategy. Despite the rising interest in TAK1 as a possible therapeutic target, earlier inhibitors of TAK1 (so-called takinibs) lacked selectivity (36). It was previously demonstrated that, distinct from other TAK1 inhibitors, HS-276 showed exquisite selectivity (13). In kinome-wide screening assays, HS-276 showed a selectivity profile of selectivity score (1) (S[1]) = 0.037 and demonstrated the most potent (IC_50_ = 2.5 nM) inhibition of TAK1 activity over all other close homologs (13). Like the takinibs, HS-276 binds in the ATP binding site of TAK1 and functions as a competitive ATP inhibitor. Biochemical assays to determine the IC_50_ are commonly performed at concentrations that are 2-fold > Km for ATP (20 μM), which is multiple-fold lower than ATP concentrations observed within cells. Due to this discrepancy in competitive ATP concentrations, we can predict and subsequently observed a shift in the IC_50_ from biochemical assays (i.e., radioactive phosphate transfer) to in vitro cell assays (i.e., 1–10 mM ATP, cytokine inhibition). Our previous studies with HS-276 demonstrated this shift and indicated that low μM HS-276 concentrations elicit robust efficacy in both cell assays and animal models (13). Our preliminary testing of a series of concentrations of HS-276 showed that 10 μM exerted potent and consistent antifibrotic effects.

While, at present, no TAK1-targeted therapies are FDA approved, limiting our understanding of how TAK1 inhibition will be tolerated in humans, a large body of preclinical work on TAK1 inhibition exists. These observations suggest that potential adverse effects of inhibiting TAK1 could mirror those seen in anti-TNF therapies, with increased risks of opportunistic diseases resulting from the systemic reduction of TNF, partially limiting the immune response to pathogens. Very importantly, unlike monoclonal antibodies against TNF, HS-276 as a small molecule has the potential to be dosed at levels that reduce, not eliminate, inflammatory signaling. By reducing maladaptive cytokine signaling to physiological levels, small molecule TAK1 inhibitor therapies may avoid adverse events observed by other cytokine-targeted therapies.

Our data provide confirmation of the previously observed upregulated TAK1 activity in SSc, and in the lungs. Moreover, these are the first results to our knowledge to demonstrate that our potentially novel, highly selective, and clinically tractable TAK1 inhibitor can protect mice from fibrosis in multiple organs. The novelty and significance of these observations lies in their obvious relevance to improving SSc outcomes. In the present study, treatment with HS-276 exerted potent antifibrotic and antiinflammatory effects on bleomycin-induced skin fibrosis that recapitulate the inflammatory stage of SSc. Moreover, the antifibrotic effects of HS-276 were not restricted to preventive application; they were also present when treatment was initiated after fibrosis had already been established. Most importantly, HS-276 exhibited marked efficacy in bleomycin-induced lung fibrosis. Besides abrogating fibrosis, HS-276 also downregulated markers of inflammation, such as expression of macrophage-specific F4/80 and inflammatory leukocyte-specific CD45. Our presented data demonstrate efficacy of HS-276 in preclinical models of organ fibrosis in preventive as well as in therapeutic settings. These results are consistent with a prior report showing that fibroblast-specific TAK1-KO mice showed impaired skin wound repair, with reduced skin thickness, collagen deposition, and myofibroblasts differentiation (20).

We found increased TAK1 activation in SSc skin and SSc-ILD lung biopsies, as well as in SSc skin fibroblasts, compared with healthy controls. Importantly, inhibiting TAK1 reduced collagen gene expressions, myofibroblast differentiation, and production of the endogenous TLR4 ligands DAMP Fn-EDA and tenascin C in SSc skin fibroblasts. We have shown previously that both of these DAMPs are markedly upregulated in SSc biopsies and are responsible for fibrosis progression (14, 15). Given the role of TAK1 in TLR4 signaling, we speculated that blocking TAK1 activity will downregulate TLR4-dependent fibrotic DAMP generation. Therefore, the antifibrotic effects of HS-276 might be, in part, attributed by reduction of profibrotic DAMPs. Consistent with our results, previous observations showed that a commercially available TAK1 inhibitor reduced the TGF-β–induced profibrotic responses in dermal fibroblasts, and fibroblast-specific TAK1-KO in mice had reduced dermal thickness, collagen deposition, and myofibroblasts differentiation (20). Together, these results indicate that selective pharmacological targeting of TAK1 activation will prevent the onset and progression of fibrosis in multiple tissues in preclinical disease models and will abrogate constitutive fibrotic responses in SSc fibroblasts in vitro. Therefore, selective TAK1 inhibition using small molecules might provide entirely new opportunities for safe and effective targeted therapy of SSc and other chronic fibrosing conditions.

## Methods

### Cell culture and reagents.

Primary fibroblast cultures were established by explantation from neonatal foreskin as well as from skin biopsies of patients with SSc and healthy adults (37). Clinical information for participants used in this study is shown in Table 1 and Table 2. Fibroblasts were grown in adherent monolayers in 100 mm plastic dishes and studied at low passage. Cultures were maintained in DMEM supplemented with 10% FBS (Thermo Fisher Scientific), 1% vitamin solutions, and 2-mM glutamine. All other tissue culture reagents were from Lonza. HS-276 (provided by EydisBio, Durham, North Carolina, USA; affiliated with Duke University) was diluted in cell culture–grade DMSO for main stock solution; working concentrations were diluted with respective media used for cell culture experiments. The control cells were treated with DMSO at concentration used with HS-276. Adult skin fibroblasts were placed in 1% FBS and incubated with HS-276 (10 μM). Foreskin fibroblasts were serum starved for 24 hours (0.1% BSA), followed by HS-276 pretreatment for 60 minutes, and treatment with TGF-β1 (10 ng/mL; Peprotech) for 24 hours. In other experiments, HS-276 was added to the cultures 24 hours after TGF-β1 stimulation. To study Smad2 activation, cultures were incubated with HS-276 for 24 hours, followed by stimulation with TGF-β1 for 60 minutes.

### Isolation and analysis of RNA.

At the end of experiments, total RNA from SSc, healthy adult, and foreskin fibroblasts was isolated and reverse-transcribed to cDNA (cDNA Synthesis Supermix; Quanta Biosciences). Products (100 ng) were amplified using SYBR Green PCR Master Mix (Applied Biosystems) on an Applied Biosystems 7500 Prism Sequence Detection System. Data were normalized to internal control GAPDH RNA and are represented as the fold change (37). Primers used are shown in Table 3.

### Bulk RNA-Seq and data analysis.

Total RNA was isolated from treated and untreated fibroblasts using quick-RNATM MiniPrep Kit from (Zymo Research). The quality of RNA was determined by using an Agilent Bioanalyzer (Agilent Technologies) and subjected to 151 bp paired-end sequencing according to the manufacturer’s protocol (Illumina NovaSeq). Differential gene expression analysis was performed with R-based differential expression package DESeq2 and iPathwayGuide software used for GO pathway analysis (Advaita Bioinformatics). Stringent statistical criteria were used to identify differentially expressed genes and pathway analysis with adjusted *P* < 0.05 (GEO accession no. GSE232435). Heatmaps for inflammatory genes were generated using GraphPad prism based on fold change values and adjusted *P* < 0.05.

### Immunoblotting analysis.

At termination of the experiments, fibroblasts were harvested and equal amounts of whole-cell lysates or culture media (15–20 μg) subjected to SDS-PAGE electrophoresis, as described (15). Membranes were incubated with primary antibodies specific for type I collagen (Southern Biotechnology, 1:1,000, 1310-01), ASMA (Sigma-Aldrich, 1:2,000, A5228), Fn-EDA (Sigma-Aldrich, 1:1,000, F6140), Tenascin-C (Abcam, 1:1,000, ab108930), phospho-Smad2 (Cell Signaling Technology, 1:400, 3108), or GAPDH (Santa Cruz Biotechnology Inc., 1:1,000, sc365062), and bands were detected using enhanced chemiluminescence (15). Band intensities were quantitated using ImageJ software (NIH) and corrected for GAPDH in each lane. See complete unedited blots in the supplemental material.

### Immunofluorescence confocal microscopy.

To assess HS-276 modulation of fibroblast responses, SSc or healthy control skin fibroblasts were incubated with HS-276 for 24 hours. At the end of the experiment, fibroblasts seeded in 8-well chamber slides were fixed with 4% paraformaldehyde or methanol, followed by incubation with 0.1% Triton-X 100. Blocking solution (5% BSA) was added, followed by primary antibodies specific to type I collagen (Southern Biotechnology, 1:300, 1310-01), phospho-TAK1 (Cell Signaling Technology, 1:100, 4531), total TAK1 (Cell Signaling Technology, 1:50, 4505), phospho-Smad2 (Cell Signaling Technology, 1:300, 18338), Smad2 (Santa Cruz Biotechnology Inc.,1:100, sc393312), Fn-EDA (Sigma-Aldrich, 1:200, F6140), procollagen 1 (Sigma-Aldrich, 1:200, Mab1912), and ASMA (Sigma-Aldrich, 1:200, A5228) at 4°C overnight, followed by incubation with respective secondary antibodies (Alexa Fluor, 1:200). After thorough washes with PBST, cells were incubated with DAPI (Sigma-Aldrich, 0.2 μg/mL) to stain nuclei, followed by mounting (38).

For immunofluorescence microscopy, healthy and SSc skin biopsies and SSc-ILD and non-SSc control lung sections were paraffin embedded, and tissue sections were immunolabeled with primary antibodies for phospho-TAK1 (Cell Signaling Technology, 1:100, 4508), CD45 (Santa Cruz Biotechnology Inc., 1:200, sc1178), ASMA (abcam, 1:200, ab5694), or F4/80 (Invitrogen, 1:200, 14-4801-82), followed by addition of respective Alexa Fluor secondary antibodies (1:200). Sections were stained with DAPI for nuclei identification and mounted with FITC mounting media (15). Images were captured using NIKON confocal microscopy (Microscopy Core, University of Michigan).

### Mouse model of skin and lung fibrosis.

Groups of 8-week-old female C57BL/6J mice (*n* = 6–8 mice/group) from The Jackson Laboratory were randomized to receive PBS, TAK1 inhibitor (HS-276, 25 mg/kg), or bleomycin (10 mg/kg) alone or combined with HS-276 (25 mg/kg) (39). Bleomycin was administered by daily s.c. injections for 10 days (5 days/week), while HS-276 (25 mg/kg) was given by daily i.p. injections starting concurrently with bleomycin for 10 days (5 days/week), followed by treatment for another week (5 days/week). Mice were euthanized on day 22, and skin and lungs were harvested for analysis. In other experiments (regression model), HS-276 (25 mg/kg) was administered starting on day 14 after bleomycin and continued until mice were harvested on day 28. In each treatment group, lungs from 4 mice were subjected to lung perfusion followed by histology and immunofluorescence assays, and lungs from the other 4 mice were subjected to hydroxyproline assays. Thus, histological examination was performed on lungs from mice distinct from those used for collagen measurement.

Paraffin-embedded skin and lung tissue sections that were 4 μm–thick were stained with H&E or trichrome or sirius red. Dermal thickness was determined by measuring the distance between epidermis-dermis junction and dermis-adipose layer junction of the skin at > 5 randomly selected sites/hpf. Lungs (left) and skin were subjected to hydroxyproline assays (Biovision). RNA was isolated from skin biopsies (Qiagen, RNeasy Plus Kit, 74134) and reverse transcribed to cDNA using Supermix for analysis by quantitative PCR (qPCR) using Applied Biosystems 7500 Prism Sequence Detection System (40).

### Statistics.

Two-tailed Student’s *t* test (unpaired) were used for comparisons between 2 groups, with *P* < 0.05 considered statistically significant. Comparisons among 3 or more groups were performed using 1-way ANOVA followed by Tukey’s multiple comparison post hoc test. Data are presented as mean ± SEM, unless otherwise indicated. Graph Pad prism (Graph Pad Software version 8, Graph Pad Software Inc.) was used for data analysis.

### Study approval.

Biopsies were performed with written informed consent, as per protocols approved by the IRB for Human Studies at Northwestern University and the University of Michigan. Clinical information of participants is mentioned in Tables 1 and 2. Animal experiments were performed according to institutionally approved protocol and in compliance with guidelines of the University of Michigan Unit for Laboratory Animal Medicine.

### Data availability.

RNA-Seq data are stored in the University of Michigan Data Den archival facility. We submitted RNA-Seq data in a GEO data set (accession code GSE232435). All the raw and processed data is stored in University of Michigan shared folder S:\Intmed_Rheum\Research\VSclero_Lab and are available upon request.

## Author contributions

S Bale, S Bhattacharyya, JV conceptualized the study. S Bale and S Bhattacharyya wrote the original draft of the manuscript. S Bale, BY, and MA performed mouse experiments and analysis. S Bale and PV performed all other experiments and data analysis. DK and PT provided skin fibroblasts. SAS, PH, and TH provided the inhibitor and helpful comments for designing the experiments. All the authors reviewed and edited the manuscript.

## Supplementary Material

Supplemental data

Supplemental data set 1

## Figures and Tables

**Table 2:**
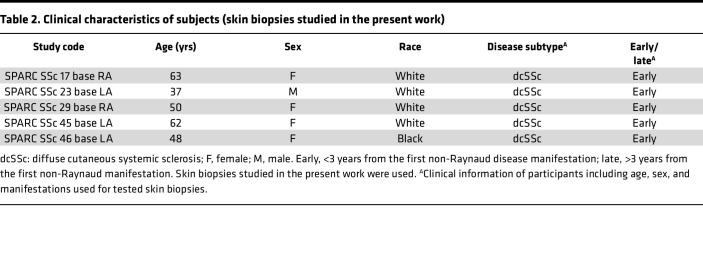
Clinical characteristics of subjects (skin biopsies studied in the present work)

**Table 1:**
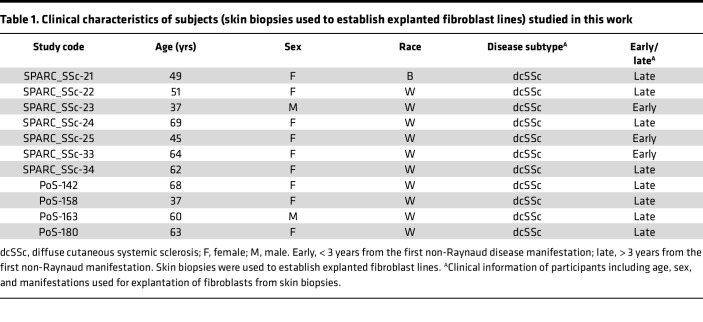
Clinical characteristics of subjects (skin biopsies used to establish explanted fibroblast lines) studied in this work

**Table 3:**
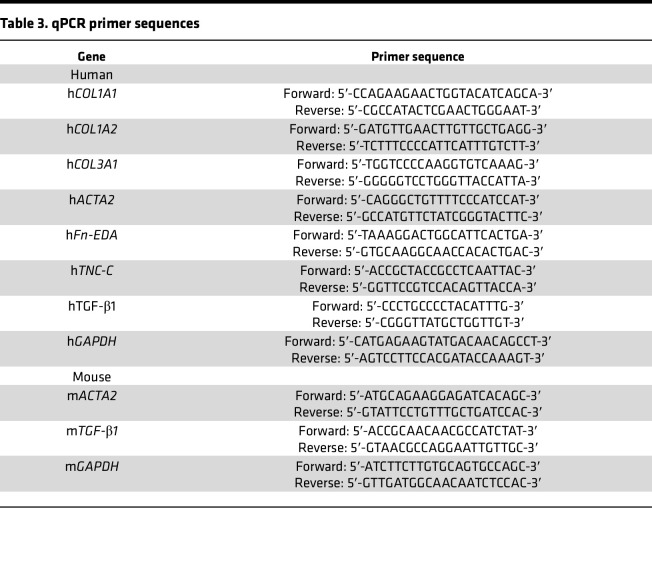
qPCR primer sequences

## Version 1 06/12/2023

In-Press Preview

## Version 2 07/24/2023

Electronic publication
