# Supplementary material for: Pharmacological inhibition of TAK1 prevents and induces regression of experimental organ fibrosis
Source: JCI Insight. 2023 Jul 24;8(14):e165358. doi: 10.1172/jci.insight.165358 (PMC10443806; doi:10.1172/jci.insight.165358)
Supplement: Supplemental data [file jciinsight-8-165358-s202.pdf]

Supplementary Figure 1

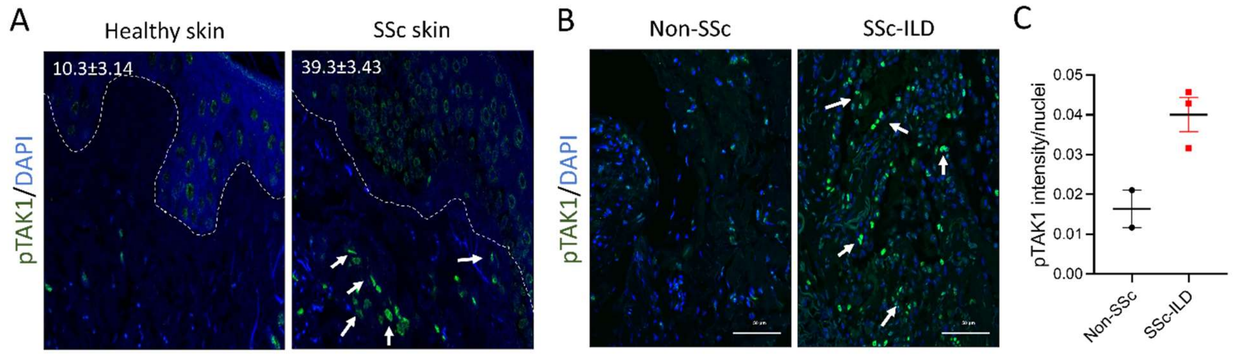

Supplementary Figure 1: TAK1 activation in SSc skin and SSc-ILD lung biopsies. A. Immunolabelling of healthy adult and SSc skin biopsies (n=5) with phospho-TAK1 antibodies (immunopositive cells/hpf). White dotted lines indicate epidermis, white arrows indicate phospho-TAK1-immunopositive cells. B. Phospho-TAK1 immunostaining of SSc-ILD (n=3), and non-SSc control (n=2) lung tissues. Representative images, scale bar-50 μm. White arrows indicate phospho-TAK1-immunopositive cells. C. Immunofluorescence intensities (means from three randomly selected regions).

Supplementary Figure 2

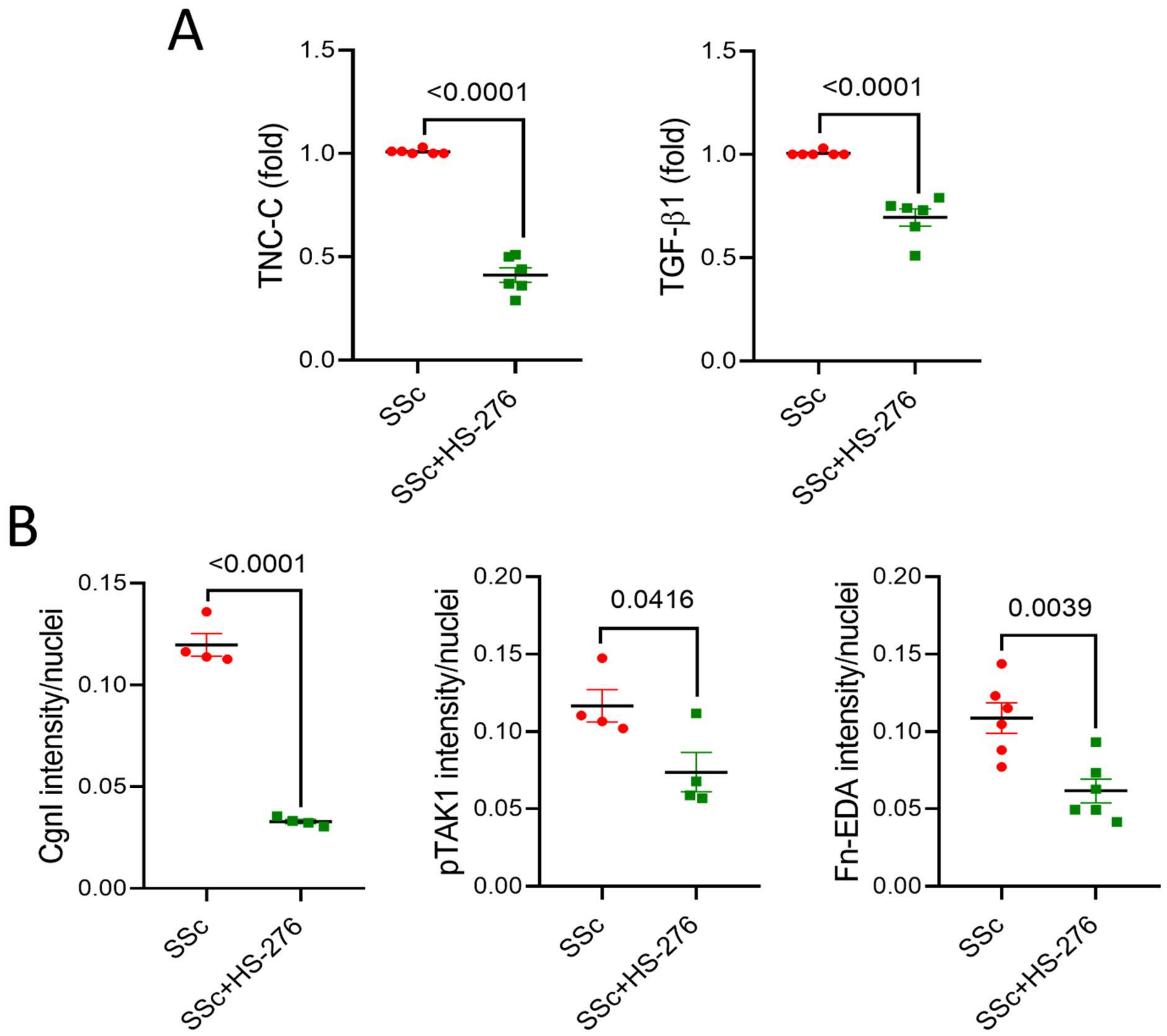

Supplementary Figure 2: Suppression of constitutive activated phenotype of SSc fibroblasts by HS-276. Confluent SSc skin fibroblasts (n=6) were incubated with HS-276 for 24 h. A. Real-time quantitative PCR, normalized with GAPDH. Unpaired t test. B. Fibroblasts were immunolabeled with antibodies to collagen I (n=4), phospho-TAK1 (n=4), and Fn-EDA (n=6). Relative fluorescence intensities. Unpaired t test.

Supplementary Figure 3

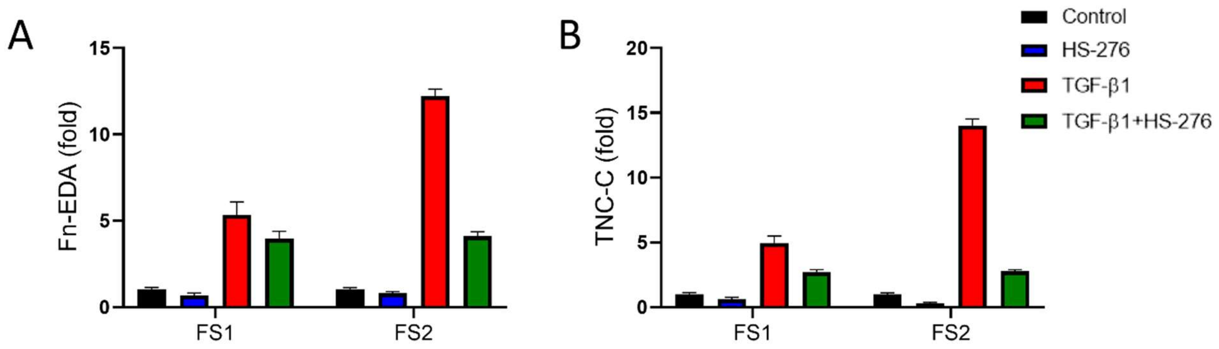

Supplementary Figure 3: HS-276 attenuated the stimulation of profibrotic DAMPs. Confluent foreskin fibroblasts were incubated with TGF-β1 (10 ng/mL) for 24 h in the presence or absence of HS-276 (10 µM). mRNA levels determined by qPCR; results normalized with GAPDH (n=2 biological replicates indicated as two independent foreskin fibroblast lines (FS)).

Supplementary Figure 4

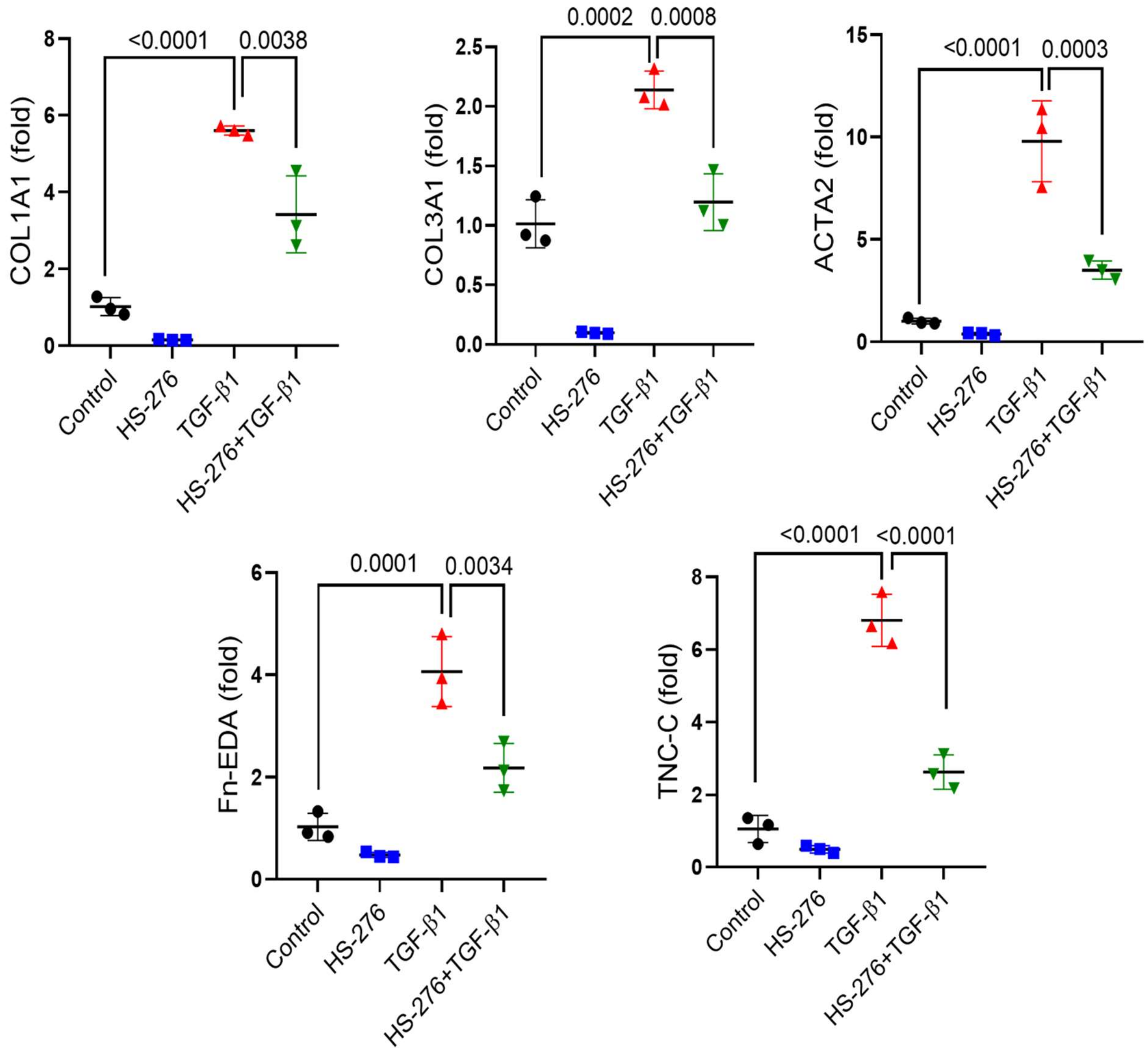

Supplementary Figure 4: Attenuation of fibrosis-associated genes in HS-276-treated healthy adult fibroblasts. Skin fibroblasts were treated with TGF-β1 with or without HS-276 for 24 h. RNA was isolated and subjected to qPCR. One-way analysis of variance followed by Tukey's multiple comparisons test.

Supplementary Figure 5

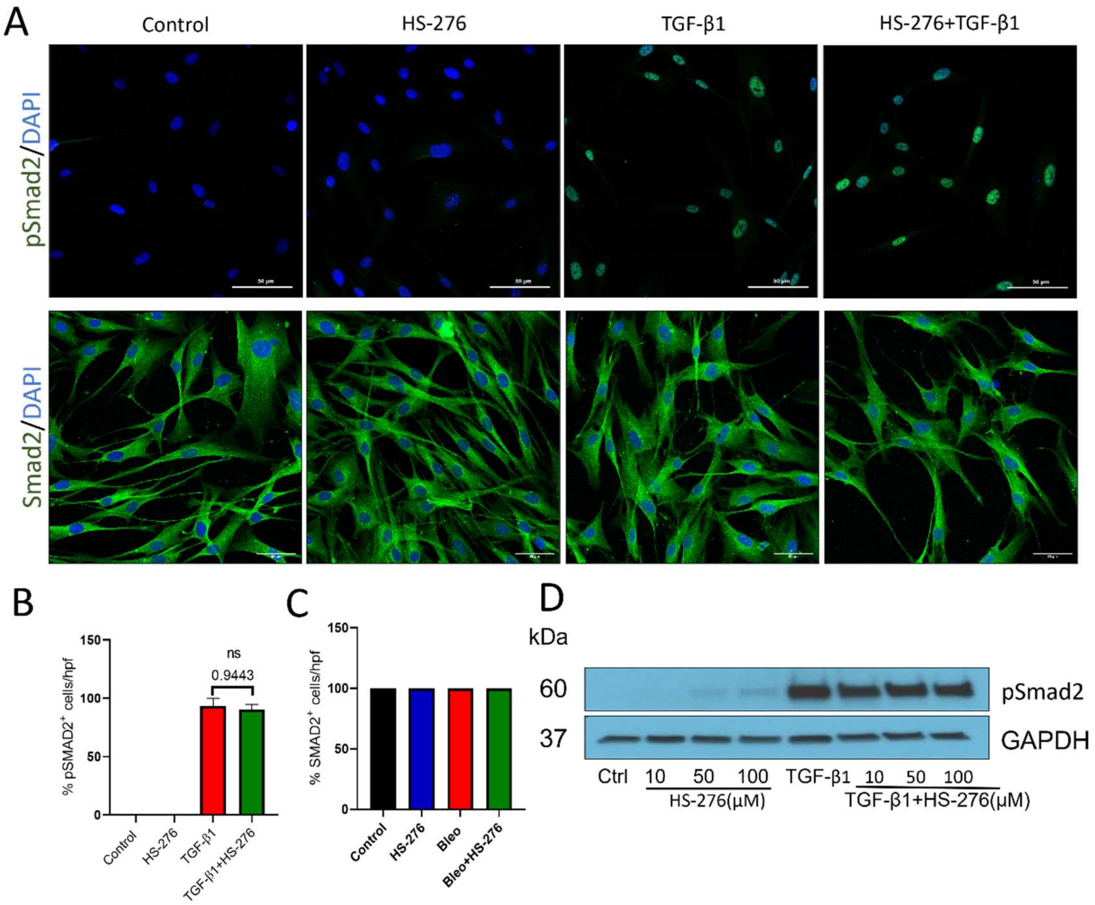

Supplementary Figure 5. HS-276 inhibits fibrotic responses independent of Smad signaling. Confluent foreskin fibroblasts were incubated with TGF-β1 (10 ng/mL) in the presence or absence of HS-276 (10 μM). A). Fibroblasts were immunolabelled with antibodies to phospho-Smad2 (top panel, green), Smad2 (bottom panel, green) and DAPI (blue). Representative images, scale bar-50 μm. B, C) Quantification of immunopositive positive cells. One-way analysis of variance followed by Tukey's multiple comparisons test. D) Whole-cell lysates examined by immunoblotting; representative blots.

Supplementary Figure 6

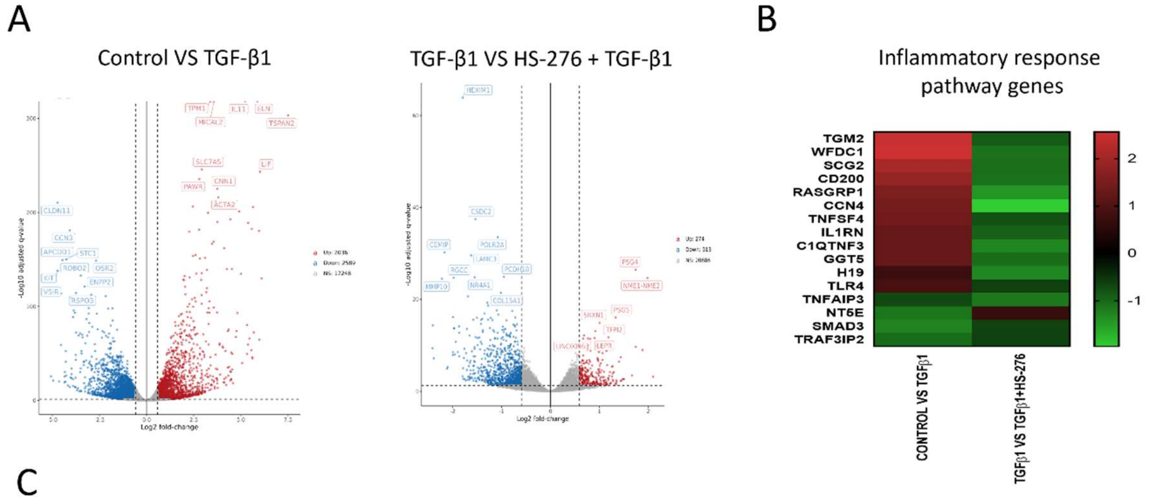

C

Name	GoId (StimDrug vs. Stim - mRNA (RNA-seq))	CountDE (StimDrug vs. Stim - mRNA (RNA-seq))	CountAll (StimDrug vs. Stim - mRNA (RNA-seq))	pv (StimDrug vs. Stim - mRNA (RNA-seq))	CountDE (Stim vs. Control - mRNA (RNA-seq))	CountAll (Stim vs. Control - mRNA (RNA-seq))	pv (Stim vs. Control - mRNA (RNA-seq))
Actin cytoskeleton organization	GO:0030036	64	568	0.009880072	248	625	1.06E-10
Canonical Wnt signaling pathway	GO:0060070	30	241	0.044536062	94	264	0.014055137
Regulation of ERK1 and ERK2 cascade	GO:0070372	27	180	0.008316342	82	213	0.002490817
Inflammatory response	GO:0006954	60	431	0.0000933	208	507	2.02E-10
Interleukin-6 production	GO:0032635	17	86	0.005214623	43	102	0.008881053
MAPK cascade	GO:0000165	63	531	0.003723789	217	601	0.0000125
Regulation of inflammatory response	GO:0050727	31	217	0.008056275	99	244	0.0000735
Regulation of smooth muscle cell proliferation	GO:0048660	21	115	0.003442033	54	126	0.001557853
Regulation of smooth muscle contraction	GO:0006940	11	38	0.002629064	23	45	0.007134868
Transforming growth factor beta production	GO:0071604	8	32	0.029715034	23	37	0.000219091

Supplementary Figure 6: Modulation of gene expression by HS-276RNA isolated from the treated and untreated fibroblast was subjected to RNA-seq. DESeq2 software was used for differential gene expression analysis and iPathwayGuide software for GO pathway analysis. Heatmaps for inflammatory genes were generated using Graphpad prism based on the fold change values and adjusted $p < 0.05$. A. Volcano plots of differentially upregulated genes (red) and downregulated genes (blue). B. Representative heatmap of inflammatory genes that are downregulated by HS-276. C. Fibrosis-related pathways related differentially regulated by HS-276.

Supplementary Figure 7

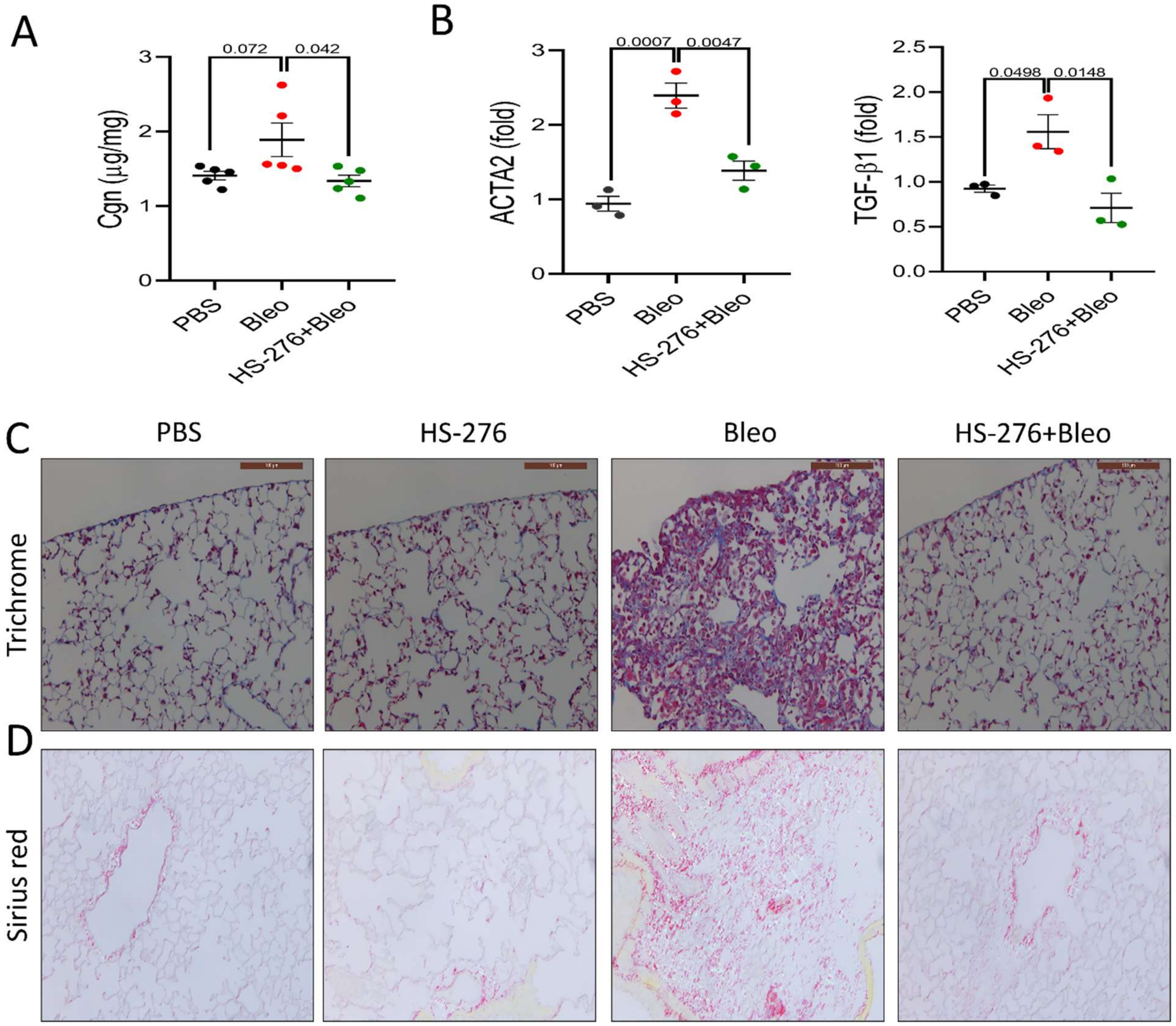

Supplementary Figure 7: HS-276 prevented bleomycin-induced fibrotic responses in skin. C57/BL6 mice were administered with daily s.c. injections of bleomycin alone or together with HS-276 (25 mg/kg, i.p.). Mice were euthanized on day 22 and skin was harvested for analysis. A. Hydroxyproline assays (n=5) B. C57/BL6 mice were randomized to four treatment groups, euthanized on day 22 and lungs were harvested. Results of qPCR (n=3). Unpaired t test. C. Trichome stain of lung tissue sections; D. Sirius red staining of lung tissue; representative images.

Supplementary Figure 8.

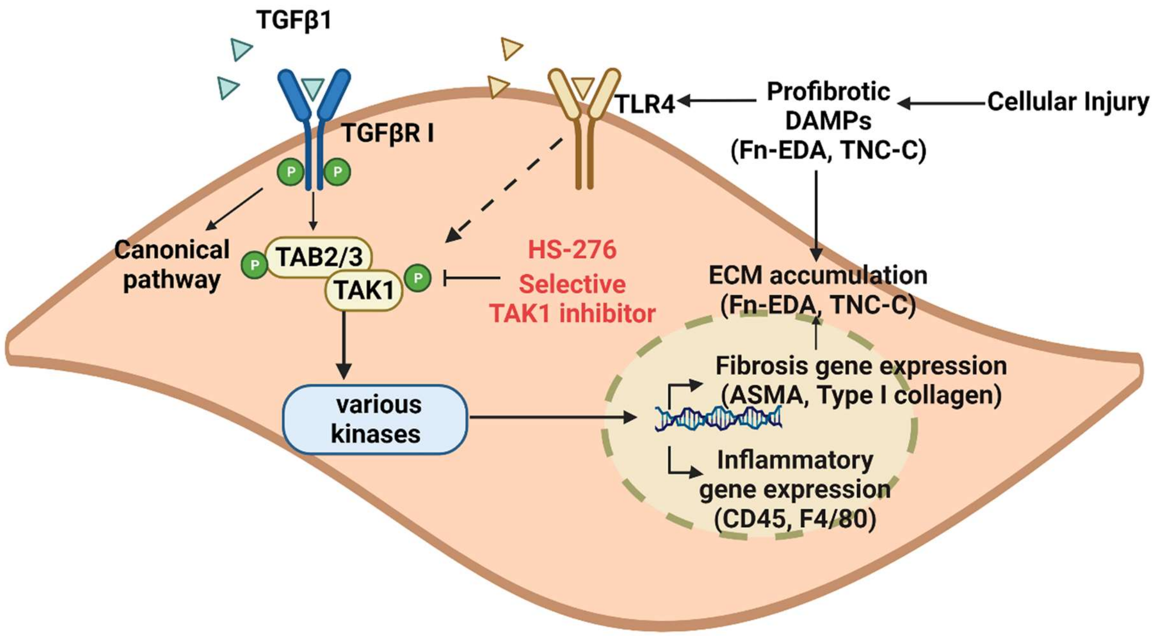

Supplementary Figure 8: Schematic illustration for the proposed mechanisms underlying the beneficial effects of TAK1 inhibition. HS-276 reduced TAK1 phosphorylation, resulting in reduced expression of fibrosis markers, including profibrotic endogenous TLR4 ligands (DAMPs).
